# Assessment of the superior photocatalytic properties of Sn^2+^-containing SnO_2_ microrods on the photodegradation of methyl orange

**DOI:** 10.1038/s41598-023-40659-8

**Published:** 2023-09-07

**Authors:** Alexandre de Oliveira Jorgetto, Maria Valnice Boldrin Zanoni, Marcelo Ornaghi Orlandi

**Affiliations:** 1 Department of Engineering, Physics and Mathematics, Institute of Chemistry, São Paulo State University (UNESP), Araraquara, SP 14800-060 Brazil; 2 National Institute for Alternative Technologies of Detection, Toxicological Evaluation and Removal of Micropollutants and Radioactives (INCT-DATREM), Institute of Chemistry, São Paulo State University (UNESP), P.O. Box 355, Araraquara, SP 14800-900 Brazil

**Keywords:** Materials for devices, Materials for energy and catalysis, Heterogeneous catalysis, Photocatalysis, Synthetic chemistry methodology

## Abstract

A microporous Sn^2+^-containing SnO_2_ material presenting microrod morphology and a surface area of 93.0 m^2^ g^–1^ was synthesized via a simple hydrothermal route. Sn^2+^ ions were detected in the interior of the material (15.8 at.%) after the corrosion of a sample through sputtering. The material’s optical properties have demonstrated the absorption of a considerable fraction of visible light up to wavelengths of 671 nm, due to the presence of Sn^2+^ states in the material’s band structure. The analysis of the internal crystalline structure of a single microrod was carried out with the aid of a focused ion beam microscope and indicated that the material is mesocrystalline down to nanoscale level. It was proposed that the Sn^2+^ ions occupy intergranular sites in the highly defective crystalline structure of the material and that Sn^2+^ states, as well as its relatively large surface area, are responsible for the material’s superior photoactivity. The synthesized material was tested as a photocatalyst to decompose hazardous contaminants in water. The photocatalytic performance of the material was much higher than those of commercial TiO_2_ and SnO_2_ materials, decomposing nearly all methyl orange (an azo-dye model) content in water (10 mg L^–1^) in 6 min under UV irradiation for a photocatalyst dose of 5.33 g L^–1^. The photodegradation of methyl orange was also verified under visible light.

## Introduction

The generation and emission of toxic substances to the environment pose a serious concern with respect to the contamination of soils, natural waters and air, due to their deleterious effects on the environmental balance and on the health of all living organisms. Among the extensive list of organic water contaminants, the azo-dyes deserve special attention. This class of compound encompasses more than 2000 substances, and their production corresponds to two thirds of the worldwide production of synthetic dyes. Such a mass manufacturing is due to their versatility, since azo-dyes meet applications in diverse products such as printing pigments, paints, varnishes, waxes, pharmaceuticals, dyes for coloring paper, leather, plastics etc.^1,2^. Thus, considering that many of these compounds—as well as their decomposition by-products, such as benzidine and other aromatic amines—are mutagenic, carcinogenic or genotoxic^2,3^, the effects of azo-dyes emission to the environment should be mitigated.

To accomplish this purpose, it is of paramount importance that the substances emitted by industrial, urban and pharmaceutical activities undergo adequate treatment prior to their release into the environment. In this respect, more sophisticate water treatment methods have been developed in the last decades, such as Advanced Oxidation Processes (*AOP*s), which aim to supplement conventional water treatments and remove persistent emerging pollutants. Among the most studied AOPs are the photo-Fenton and Fenton processes, photolysis, ozonolysis, photocatalysis and photoelectrocatalysis. As a common feature, these processes share the generation of reactive oxygen species (hydroxyl radical, peroxide radical, hydrogen peroxide etc*.*) in the medium. Since such species are strong oxidants exhibiting low selectivity, they react with most of the organic compounds in aqueous media, driving their decomposition to CO_2_, H_2_O and their constituent minerals (*mineralization*)^4–6^. Concerning photocatalysis (which consists of a heterogeneous type of catalysis), the decomposition of pollutants is possible due to the transition of electrons in the electronic structure of semiconducting materials. To be more specific, electrons from the valence band (*VB*) are promoted to the conduction band (*CB*) upon irradiation of the material with electromagnetic radiation of sufficient energy (energy of the photons equals or is greater than the band gap energy of the semiconductor). According to this mechanism, both photoexcited electrons in the CB and photoexcited holes in the VB may migrate through the material to drive redox reactions at its surface^5,7^. Then, at the interface between the semiconductor surface and the medium, adsorbed contaminant molecules can be directly oxidized by the holes. Similarly, water and oxygen molecules may also undergo interfacial redox reactions with the photoexcited electrons and holes to form reactive oxygen species, which diffuse into the medium and induce radical reactions with contaminant molecules, ultimately leading to their mineralization.

TiO_2_ is one of the most successful semiconductor material in the field of photocatalysis^6,8^, due to its high photodegradation performance under UV light, chemical and physical stabilities, abundance and non-toxicity^6,9^. Although the photocatalytic properties of TiO_2_ have been extensively demonstrated, its large band gap energy (*E*_g_ ~ 3.3 eV) has frequently being pointed out as a setback for its practical application in solar-light-driven photocatalysis^7,10–13^, because pristine TiO_2_ can only absorb UV light (a minor fraction of the solar spectrum), therefore hindering the efficient harnessing of the abundant and eco-friendly visible light provided by the sun. Similarly, SnO_2_ has also been employed in photocatalysis and suffers from the same setback of TiO_2_, since it presents a band gap energy of around 3.6 eV, being strictly able to photodegrade organic contaminants under UV-light irradiation, when in its pristine form^14^. To circumvent such a limitation, research groups have modified TiO_2_ and SnO_2_ through doping^7,9,11,14–16^ and the synthesis of TiO_2_- and SnO_2_-based heterostructures^14, 16–18^, aiming to obtain visible-light-responsive materials. Alternatively, other naturally visible-light-responsive semiconducting materials have been synthesized for photocatalysis, including Bi_2_O_3_^19^, Bi_2_O_4_^20^, BiOBr^21^, BiVO_4_^22^, Bi_2_MoO_6_^23^, Bi_2_WO_6_^24^, graphitic C_3_N_4_^23^, Ag_3_VO_4_^25^, NiMoO_4_^26^ and CdS^27^, but all of them present photocorrosion effects, low stability or low degradation efficiency.

In this work, a SnO_2_ material containing Sn^2+^ ions (SnO_2_/Sn^2+^) was synthesized via a simple hydrothermal route, then to be tested as a photocatalyst under UV/visible light irradiation conditions. The new material had its morphology, crystal structure, chemical composition and optical properties characterized. The photocatalyst performance of the SnO_2_/Sn^2+^ material was tested in terms of the photodegradation of methyl orange, as an azo-dye contaminant model in water.

## Materials and methods

### Reagents and materials

SnCl_2_⋅2H_2_O (J.T. Baker; ~ 100%) and trisodium citrate dihydrate (Synth; 99%) were used in the synthesis of the SnO_2_ material containing Sn^2+^ ions. The methyl orange (*MO*) dye (85% purity) was obtained from Sigma-Aldrich. All solutions were prepared with deionized water. The TiO_2_ and SnO_2_ materials used in the photodegradation experiments were both purchased from Sigma-Aldrich with the respective purities of 99–100.5% and > 99%.

### Synthesis of the SnO_2_/Sn^2+^ material

1.30 g of SnCl_2_⋅2H_2_O and 1.50 g of trisodium citrate dihydrate were stirred for 10 min in 75 mL of deionized water and then sonicated for 5 min, yielding a white suspension, which was then transferred to a Teflon hydrothermal reactor. After properly sealed, the reactor was heated to 180 °C for 2 h in a microwave oven, and then cooled naturally up to the ambient temperature. The reaction provided a yellow precipitate, and the supernatant was carefully drained with the aid of a dropper. The material was agitated in 15 mL of deionized water, centrifuged and the supernatant disposed to remove residual reaction products. This washing process was repeated 5 times. Finally, the material was dried at 70 °C and stored in a sealed flask at ambient condition prior to use.

### Equipment and characterization methods

High-resolution scanning electron microscopy (*SEM*) images were collected in a JEOL Field-effect electron microscope, model 7500F. The x-ray photoelectron spectrometry (*XPS*) analysis was carried out in a spectrometer Omicron Sphera with 7 channeltrons. Since this technique can only probe the chemical composition within few nanometers deep in the surface of samples, the microrods underwent a sputtering process with Ar^+^ ions, so to expose its interior, before the collection of the photoelectron spectra. The peaks of Sn 3*d*_3/2_ and 3*d*_5/2_, O 1*s* and C 1*s* were collected in high-resolution. The C 1*s* peak from adventitious carbon was used as reference for the calibration of the spectra, being set at 285 eV. Diffraction patterns were collected in a diffractometer model D8 Advance Eco from Bruker. The radiation used was the line Kα from Cu, and the equipment was operated at 25 mA and 40 kV. The 2*θ* range was 5 to 90°, being scanned at angular steps of 0.02°. The acquisition time at each step was 0.21 s. The micro-Raman spectroscopy analysis took place at a spectrometer model Lab RAM HR from Horiba Jobin Yvon using a laser with a wavelength of 633 nm. The Raman shift range varied from 100 to 1500 cm^–1^ at a resolution of 0.3 cm^–1^. High-resolution transmission electron microscopy (*HR-TEM*) images were collected in a FEI microscope, model Tecnai G^2^ F20, at an acceleration voltage of 200 kV. Prior to the TEM analysis, a single microrod of the sample was deposited on a Si substrate and had part of its surface coated by protective C and Pt layers in a focused ion beam (*FIB*) microscope (FEI, model Helios Nanolab 600I). Then, the ion beam of the microscope was used to thin out the SnO_2_/Sn^2+^ microrod until a longitudinal section of it was obtained (Supplementary Fig. S1a and b—in the Supplementary Material), which was later welded to a FIB lift-out grid (Supplementary Fig. S1c). Once the microrod section was immobilized at the FIB lift-out grid, it was further thinned out (Supplementary Fig. S1d) so to enable probing both the interior and the subsurface of the microrod (in the region opposite to the C and Pt layers) by TEM. The material’s specific surface area was determined through the Brunauer–Emmett–Teller (*BET*) method using the nitrogen adsorption–desorption data collected at 77 K in an ASAP 2010 equipment from Micromeritics. The porosity of the material was evaluated in terms of the Barret-Joyner-Halenda (*BJH*) method using the data from the desorption branch. The UV–Vis diffuse reflectance spectroscopy (*UV–Vis DRS*) analysis was carried out in a spectrophotometer Lambda 1050 (Perkin Elmer) equipped with accessories for reflectance measurements. The assessment of the MO content and of the intermediate species in the aqueous samples were performed at negative mode in a UV–Vis spectrophotometer Cary 60 from Agilent Technologies and in a liquid chromatography equipment (model Accela from Thermo) hyphenated with LCQ-Fleet mass spectrometers from Thermo (*LC–MS/MS*).

### Photodegradation experiments

The photodegradation experiments were carried out in a closed photocatalytic reactor containing a multi-position stirrer and light lamps positioned inside the reactor (top part). The photodegradation tests were carried out using a stock solution of 10 mg L^–1^ MO dye, previously aerated during 30 min. Afterwards, 20 mg of a material were added into 30 mL of the dye solution, remaining under stirring for 90 min to reach the adsorption equilibrium of MO dye. Samples were collected at the end of the dark regime, and then, sequentially, under illumination regime at pre-determined times, and stored in 50-mL Falcon tubes. The tubes were centrifuged at 10,000 RPM, and the supernatants were collected for analysis via UV–vis spectrophotometry. The experiments were carried out under a controlled temperature of 20 °C, under continuous air bubbling. Two types of lamps were employed in the experiments: 18 W black light lamps (Philips; TL-D BLB), and 20 W white light lamps (Philips; T10 plus).

## Results and discussion

### Morphological and surface characteristics of the SnO_2_/Sn^2+^ material

**Figure 1:**
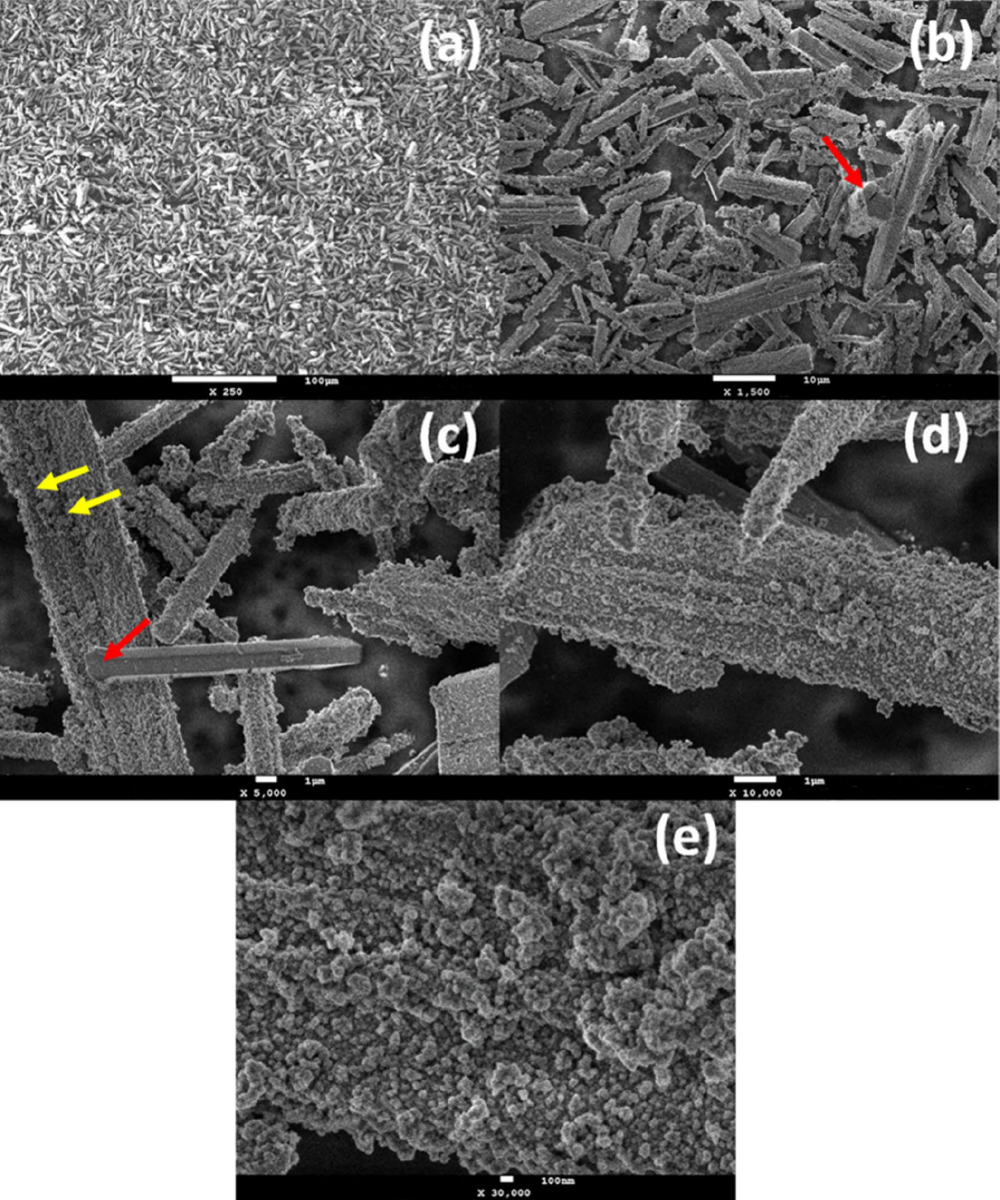
SEM images of the synthesized material exhibiting (**a**) an overview of the microrods’ morphology and size distribution, (**b**) and (**c**) the hexagonal cross section of the particles and their layered nature, (**d**) the surface texture of a microrod and (**e**) a close-up of its surface.

Figure 1 exhibits the morphological features of the synthesized material. The images (a) and (b) of this figure indicated that the hydrothermal synthesis method induced the strict formation of microrods, which tended to exhibit hexagonal cross sections, as noted from the red arrows shown in Fig. 1b and c. The preponderant widths of the microrods were of few micrometers, while their lengths extended to tens of micrometers. The size distribution of the particles showed that 89% of the particles resided within the length range from 6 to 18 µm, whereas 94% of them present widths from 0.7 to 4.2 µm (Supplementary Fig. S2a and b, respectively). Nevertheless, lengths and widths of nearly 30 and 7 µm, respectively, were also observed. The majority of the particles presented a very rough surface, although it was also possible to detect few particles with smooth surface (Fig. 1c). It was also possible to observe a layered structure along the length of some particles (yellow arrows in Fig. 1c). Figure 1d and e present magnified views of a microrod, where it is possible to verify its rough texture. Furthermore, Fig. 1e suggests that nanometric spherical particles coalesced to originate the structures of the microrods. Additionally, evidence of grain boundaries could be verified at the surface of the particles by SEM analysis carried out at very high magnifications (100 kx and 500 kx). The Supplementary Fig. S3a demonstrated that even a “smooth surface” microrod exhibited plenty of irregularities in its texture (suggesting that the microrods may not be monocrystalline), while Supplementary Fig. S3b indicated the approximate size of such grains extend up to tens of nanometers. The fact that they strictly formed microrods with hexagonal cross sections (such as in a monocrystal) suggests that these microstructures might be mesocrystalline.

To determine the material’s specific surface area and porosity, the nitrogen isotherms found in Supplementary Fig. S4a were collected. As can be noted from this figure, the material provided type-II isotherms with no evident hysteresis between the adsorption and desorption branches. This type of isotherm is typically assigned to non-porous materials^28^; however, the BJH method allowed verifying the presence of pores with average diameter of 4.0 nm and also with dimensions smaller than 2 nm (Supplementary Fig. S4b), thus demonstrating the material’s microporous nature. Furthermore, its BET specific surface area was determined as 93.0 ± 1.5 m^2^ g^–1^, which is considerably high. Therefore, the results from nitrogen adsorption demonstrated that the material’s surface properties are suitable for catalytic applications, considering the availability of adsorption sites provided by its relatively high surface area and porosity.

### Chemical composition

**Figure 2:**
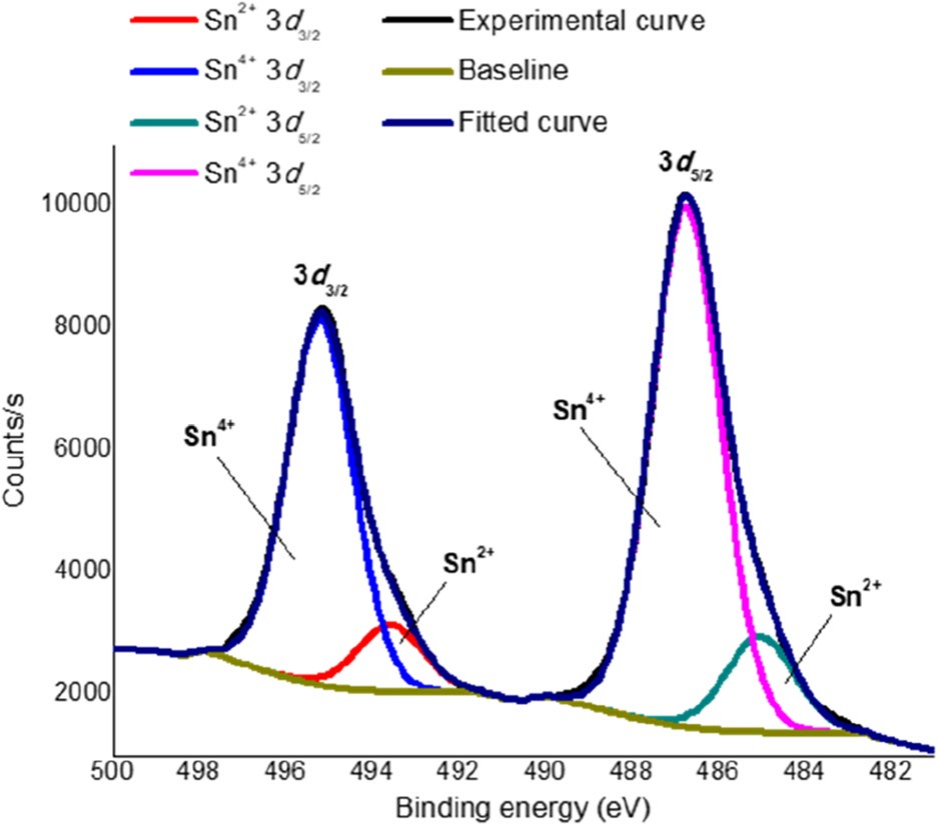
XPS spectrum of the Sn 3*d*_3/2_ and Sn 3*d*_5/2_ peaks for the synthesized material after the sputtering process.

To elucidate the chemical composition of the produced material, XPS analysis was performed and demonstrated that the material is composed by Sn, O and C, as noted from Supplementary Fig. S5a. By deconvoluting the peaks of each of these elements, it was verified that the Sn 3*d*_3/2_ and Sn 3*d*_5/2_ peaks were composed by two components each, as illustrated in Fig. 2. Whereas the curves centered at 495.20 and 486.75 eV were ascribed to Sn^4+^ states, those at 493.58 and 485.02 eV were associated to Sn^2+^ states. The integration of the areas under each component curve allowed us determining the Sn^2+^:Sn_total_ atomic ratio as 0.158, indicating that the produced material contained a considerable amount of Sn^2+^ in its interior, despite exhibiting the crystalline structure of rutile SnO_2_ (stoichiometrically composed by Sn^4+^), as discussed in "Crystalline structure" section. We believe that the presence of citrate ions in the synthesis medium partially prevented the oxidation of Sn^2+^ from the tin precursor into Sn^4+^ in SnO_2_, provided that citrate possesses reducing properties, being employed as a reducing agent in the synthesis of metallic Ag nanoparticles^29^. The deconvolution of the O 1*s* spectrum provided three component curves centered at 530.46, 531.15 and 533.46 eV, which were respectively assigned to oxygen atoms in the SnO_2_ lattice, to oxygen species exposed by the sputtering process, and to H_2_O or hydroxides (Supplementary Fig. S5b)^30^. According to the literature, the O 1*s* component at 531.15 eV should be ascribed to chemisorbed oxygen species^30^. Nonetheless, the SnO_2_/Sn^2+^ material had its surface corroded prior to the XPS analysis; therefore, we assigned this component to lattice oxygen species exposed by the sputtering process. Lastly, the C 1*s* peak was assigned solely to adventitious carbon, with contributions from C–C and C–O at 285 and 286 eV, respectively (Supplementary Fig. S5c)^31^.

The surface of the SnO_2_/Sn^2+^ material with a non-sputtered surface was also characterized by XPS. The survey of its external surface provided the same composition as noted for its interior, exhibiting peaks of Sn, O and C (Supplementary Fig. S5d). Nonetheless, the high-resolution spectra of these elements presented some discrepancies in comparison with the internal composition. With respect to the Sn 3*d* peaks (Supplementary Fig. S5e), it was noted that their deconvolution led to a smaller contribution from the Sn^2+^ ions (Sn^2+^:Sn_total_ ratio = 0.043), in comparison with the results of the sputtered microrods. We believe that this observation is due to the further oxidation of the Sn^2+^ ions at the material’s surface by air after the synthesis. Concerning the O 1*s* peak (Supplementary Fig. S5f), its total area was found higher than that measured for the sputtered sample, also pointing out to a more oxidized surface. Moreover, while the area of the oxygen atoms at the lattice remained roughly the same, the areas of the oxygen atoms related to chemisorbed species and to H_2_O/hydroxides were found greater, in comparison with the corresponding contributions obtained for the sputtered sample. Finally, concerning the C 1*s*, an extra contribution assigned to carbonate was noted at 289 eV (Supplementary Fig. S5g), which may be due to the oxidation of the carbon compounds at the surface of the material, as well as to residual citrate from the synthetic medium. All these results demonstrated that the surface of the material is at a more oxidized state than its interior, consisting of a reasonable observation.

### Crystalline structure

**Figure 3:**
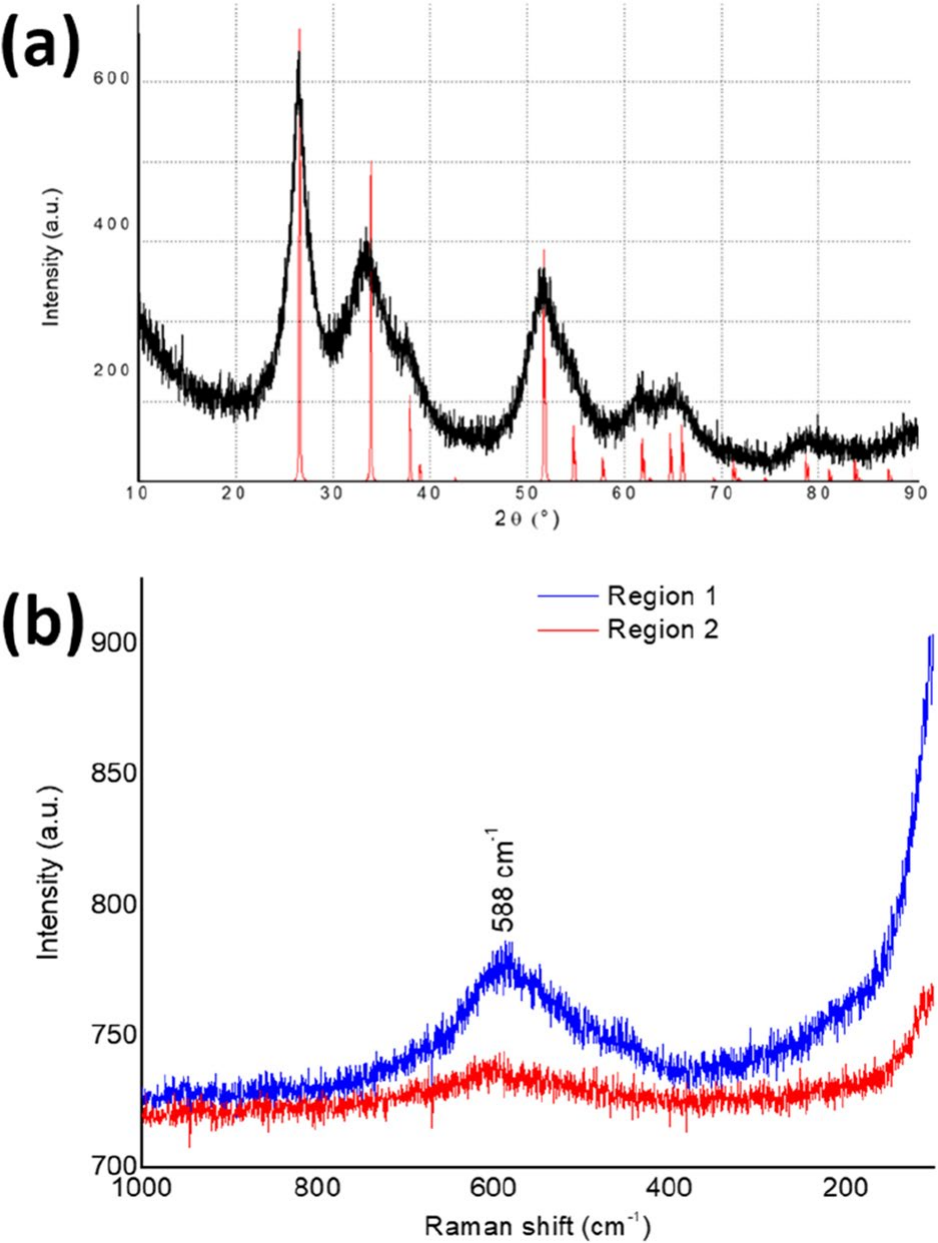
Results of the XRD (**a**) and Raman spectroscopy (**b**) analyses for the synthesized material. The Raman spectra were collected in two different regions for the same sample.

The x-ray diffractometry (*XRD*) analysis of the synthesized material was carried out, and the collected diffractogram can be seen in Fig. 3a. Along with the experimental diffraction patterns (in black), one can also find the standard diffraction pattern of tetragonal rutile SnO_2_ (PDF #41-1445—in red), whose space group is *P*4_2_/*mnm* (no. 136). As can be noted, nearly all rutile SnO_2_ standard peaks demonstrated a good correspondence with the diffraction peaks observed for the synthesized material concerning both, position and intensity, therefore indicating the crystalline structure of rutile SnO_2_ for the synthesized material.

**Figure 4:**
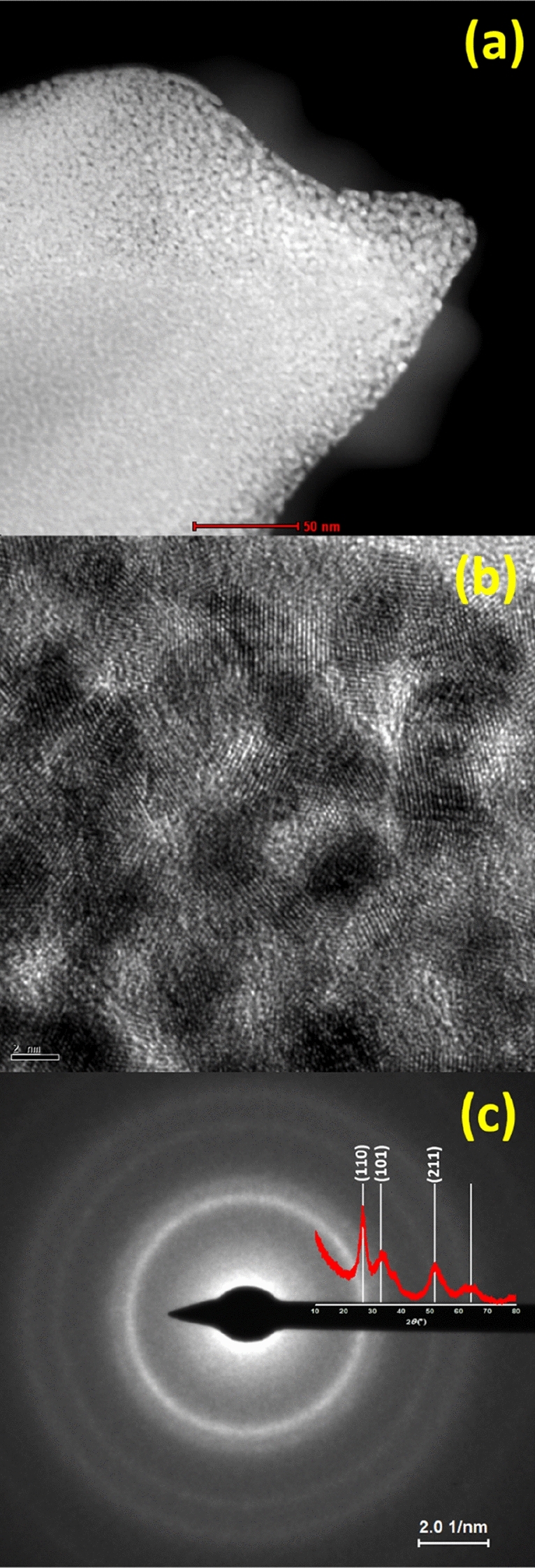
Dark field TEM (**a**) and HR-TEM (**b**) images along with the SAED analysis **(c)** of the internal structure of a SnO_2_/Sn^2+^ microrod.

It is worth noting that, although the individual particles of the material exhibited micrometric sizes, the diffraction peaks observed for the synthesized material were considerably broad. This observation indicates that the microrods are constituted by numerous nanosized grains. To ascertain such assumption, we employed HR-TEM to probe the crystalline features at the subsurface of a microrod and in its interior. To do so, a section of a single microrod was extracted and thinned out in a FIB microscope (as described in detail in the “Materials and methods” section), and then this section of the microrod was analyzed via HR-TEM, whose results can be found in Fig. 4. In Fig. 4a, the dark field image of the internal part of a microrod shows numerous bright dots caused by the diffraction of the electron beam of the microscope. Each of these dots correspond to the constituent crystal grains of the microrod. In the Supplementary Fig. S6, there is the corresponding bright field image of the same region, where it is possible to visualize the constituent grains of this single microrod. As can be noted, the grains presented sizes of few nanometers and demonstrated no well-defined shape and organization.

As one analyzes the HR-TEM image in Fig. 4b, it is possible to observe several crystalline planes extending for few nanometers, which exhibited varied orientation. The interplanar distance measurements of these planes provided ~ 2.1 Å, in agreement with the distance reported for the planes (210) of rutile SnO_2_ phase (PDF #41-1445).

Additionally, according to Fig. 4c, the selected area electron diffraction (*SAED*) analysis provided well-defined ring patterns, as it is characteristic of polycrystalline materials. These rings are evidenced by the superposition of the SnO_2_/Sn^2+^ x-ray diffractogram and could be indexed to the planes (110), (101) and (211) of rutile SnO_2_. The *d*-spacing between crystalline planes were determined with basis on the diffraction pattern of the rings in Fig. 4c, providing distances of 3.01, 2.68 and 1.86 Å for these respective planes, whereas, according to PDF#45-1445, the corresponding distances are 3.35, 2.64 and 1.76 Å. Thus, a 10-% decrease was noted for the (110) interplanar distance. In the Supplementary Fig. S7a, it is represented the tetragonal crystalline structure of rutile SnO_2_, along with the planes (110) and (1–10). As can be observed from these planes, the atomic density along the first plane is greater than that of the second, therefore, a contraction of the unit cell along the direction [110] is more probable, as represented by the blue arrows in this figure. Besides, the decrease of the interplanar distances between the planes (110) could reflect in the dilation of the unit cell in other directions, and, in fact, the interplanar distance between the planes (211) increased ~ 5%. Therefore, the angles of the originally tetragonal unit cell must have changed to values other than 90°, becaming either monoclinic or even triclinic. This effect is represented in the Supplementary Fig. S7b, as seen from the top of (001) plane. As can be verified in the Supplementary Fig. S8, the angles found in the SnO_2_/Sn^2+^ microrods exhibit an approximate correspondence with those estimated for a distorted SnO_2_ unit cell.

This discussion on the morphology of the microrods suggests that their growth process in the reaction medium was similar to that of a monocrystal (nucleation followed by crystal growth), rather than by the oriented attachment^32^ of single grains to give rise to the mesocrystalline structure noted for the synthesized material. As could be noticed from the SEM images of the material, some microrods containing no spherical particles attached to their surfaces could be found among the particles of the material. Then, it is possible that all mesocrystalline microrods initially grew rapidly in the form of smooth surface microrods, when the tin concentration was still high, from the condensation of the hydroxylated tin ions (*tin precursors*) formed from hydrolysis reactions with tin cations. As soon as most of the tin precursors were consumed during the growth of the microrods, the kinetics of formation of the tin oxide decreased, and the residual tin precursors began to originate the nanospheres from the surface defects on the microrods to attribute them their characteristic rough texture. Concerning the role of the citrate, it is reasonable to believe that it acted as a capping agent, posing a steric hindrance after its preferential adsorption on favorable facets of the growing microrods, then inhibiting their growth in this particular direction, while directing their growth via facets which the citrate adsorbed at a smaller extent^32,33^. A schematic representation of their growth can be found in the Supplementary Fig. S9.

Still concerning the TEM analysis, images were also collected at the subsurface of the material and provided analogous results, as demonstrated in Supplementary Fig. S10a to c. The polycrystalline character of this material was also demonstrated for a single nanosphere found at the surface of a microrod via SAED (Supplementary Fig. S11).

A Raman spectroscopy analysis of the material was also performed to check for surface impurities, such as SnO and Sn_3_O_4_ (natively Sn^2+^-containing phases), and the collected spectra can be found in Fig. 3b. As one can note, the Raman spectra of the synthesized material exhibited only a broad band between 400 and 700 cm^–1^, where lie the peaks associated to SnO_2_^34^. This band showed a maximum at 588 cm^–1^, indicating the presence of in-plane oxygen vacancies in the SnO_2_/Sn^2+^ material^35^. The presence of O vacancies are expected in highly-defective crystalline structures, and, in the case of the SnO/Sn^2+^ material, the defects can be ascribed to the Sn^2+^ cations residing in the SnO_2_ crystalline structure. The fact that individual well-defined characteristic SnO_2_ peaks could not be identified further corroborates that the materials demonstrated low crystallinity, in alignment with the interpretation of the XRD, TEM and SAED results. Moreover, no peaks associated to SnO and Sn_3_O_4_ were detected between 100 and 350 cm^–1^, thus supporting the high purity of the produced material.

**Figure. 5:**
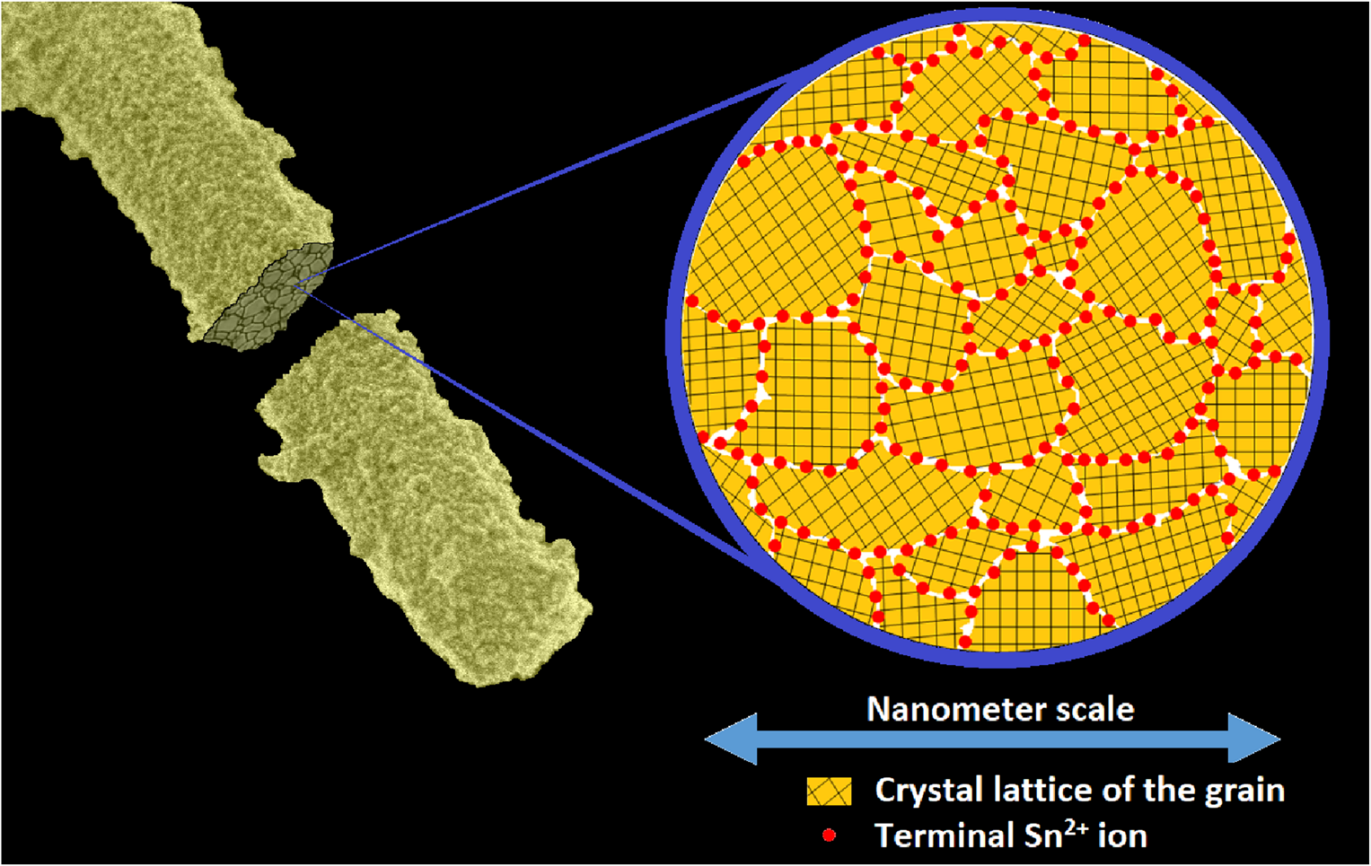
Schematic representation of the internal structure of a SnO_2_/Sn^2+^ microrod.

In summary, as it was possible to verify from the XRD, Raman spectroscopy, TEM and SAED results, the synthesized material presented a strong polycrystalline character down to nanoscale level. In addition, if any Sn^2+^-containing phases, such as SnO or other intermediate tin oxide phases (Sn_2_O_3_, Sn_3_O_4_ etc.), coexisted with the SnO_2_ phase, they should have been detected by Raman spectroscopy even at very low concentrations, since these phases scatter light more efficiently than SnO_2_^36^. Therefore, we propose that the Sn^2+^ ions detected in the interior of the SnO_2_/Sn^2+^ material through XPS analysis induced the formation of its highly defective internal crystalline structure, as schematically represented in Fig. 5. According to this figure, the SnO_2_/Sn^2+^ microrods are composed by numerous nanosized crystallites composed by Sn^4+^ ions, while the Sn^2+^ ions reside at the grain boundaries of the crystallites (as terminal cations at the edges of their lattices), where they act disrupting the long-range periodicity of the SnO_2_ crystalline structure. Based on this model, it is expected that the smaller the average size of the constituent crystallites, the greater the Sn^2+^ content to be accommodated within the grain interfaces, thus justifying the relatively large Sn^2+^ content in the interior of the synthesized material. This model may also justify the presence of the micropores in the material, as noted from the surface area measurements (“Morphological and surface characteristics of the SnO_2_/Sn^2+^ material” section).

### Opto-electronic properties

**Figure 6:**
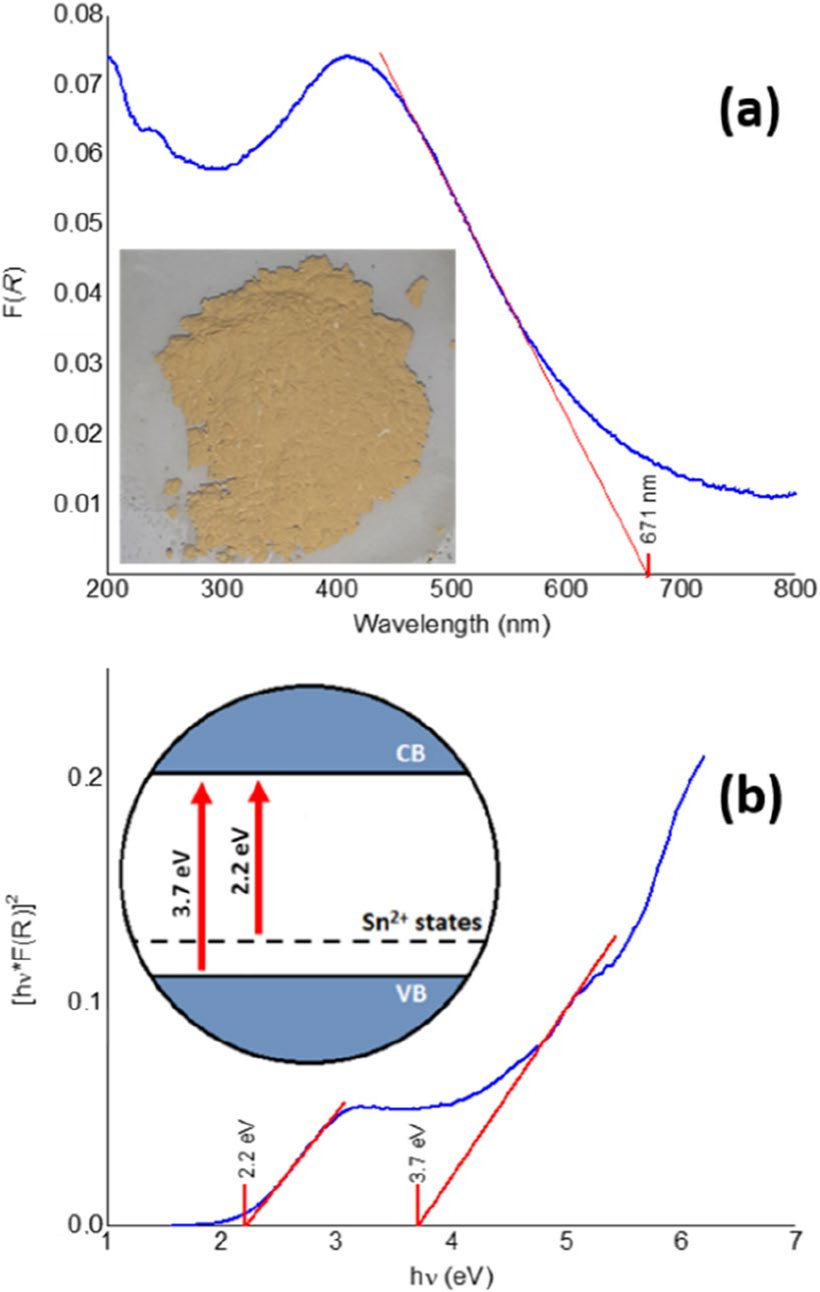
Kubelka–Munk (**a**) and Tauc (**b**) plots for the SnO_2_/Sn^2+^ material. The inset in (**a**) is a picture of the synthesized material, whereas that in (**b**) represents its band structure.

As it is reported, due to the high band gap of SnO_2_ (~ 3.6 eV), this phase is transparent to visible light^37^, nonetheless, once in powdered form (and provided that its particles are sufficiently small), it becomes white due to the scattering of ambient visible light. Contrastingly, the obtained material was evidently yellow, as shown in the embedded picture of Fig. 6a, indicating that the presence of Sn^2+^ ions altered the absorption properties of SnO_2_. To assess the optoelectronic properties of the SnO_2_/Sn^2+^ material, we employed UV–vis DRS, and the results, expressed as Kubelka–Munk and Tauc plots, are provided in Fig. 6a and b, respectively.

According to the Kubelka–Munk plot (Fig. 6a), the SnO_2_/Sn^2+^ material possesses a strong absorption within the visible-light spectral range, presenting a maximum at ~ 410 nm and an absorption edge at 671 nm. Since it is theorized that SnO_2_ possesses a direct band gap^38,39^, we estimated the direct band gap of the SnO_2_/Sn^2+^ material by means of the Tauc plot, as shown in Fig. 6b. According to the intersection of the red lines extrapolated to the *x* axis in this figure, the slope indicating 3.7 eV is in good agreement with the band gap energy of ~ 3.6 eV reported for undoped SnO_2_^40^. Nevertheless, another slope can be distinguished pointing to 2.2 eV, suggesting that the SnO_2_/Sn^2+^ material behaves as a self-doped material, and that its enhanced visible-light absorption is related to Sn^2+^ defect states introduced into the band structure of SnO_2_. The band structure of the SnO_2_/Sn^2+^ material is represented in the embedded diagram of Fig. 6b, which is based on theoretical calculations for Sn^2+^-containing tin oxide phases^38, 41^. According to these calculations, the presence of Sn^2+^ cations in SnO and mixed-valence tin oxide phases induces an upshift of their VB maximum and a consequent redshift in their photoabsorptive response, relative to pristine SnO_2_¸ due to the filling of extra Sn *s* and *p* states with the additional electrons from Sn^2+^. Such predictions were supported by Tanabe et al.’s results^42^, which determined a shallower VB maximum for Sn_3_O_4_ (+ 2.5 V *vs.* Standard Hydrogen Electrode—*SHE*), compared with that determined for SnO_2_ (+ 3.6 V *vs.* SHE).

For comparison, we also analyzed the optical properties of commercial SnO_2_. To ensure that the Sn^2+^ content in this material was totally oxidized into Sn^4+^, it was annealed in air at 1000 °C for 2 h. According to the Kubelka–Munk and Tauc plots obtained for this material (Supplementary Fig. S12a and b, respectively), the SnO_2_ sample exhibited an absorption edge at 340 nm, with a band gap energy of 3.8 eV, in alignment with the values found in the literature for SnO_2_^40^. This result evidenced that SnO_2_ containing virtually none Sn^2+^ ions cannot use visible light to drive photocatalytic processes, in contrast with the material SnO_2_/Sn^2+^. It is worth highlighting that such enhanced absorption of visible light may promote the application of the SnO_2_/Sn^2+^ material in solar-driven photocatalysis and photosensing, for instance.

### Photodegradation experiments

**Figure 7:**
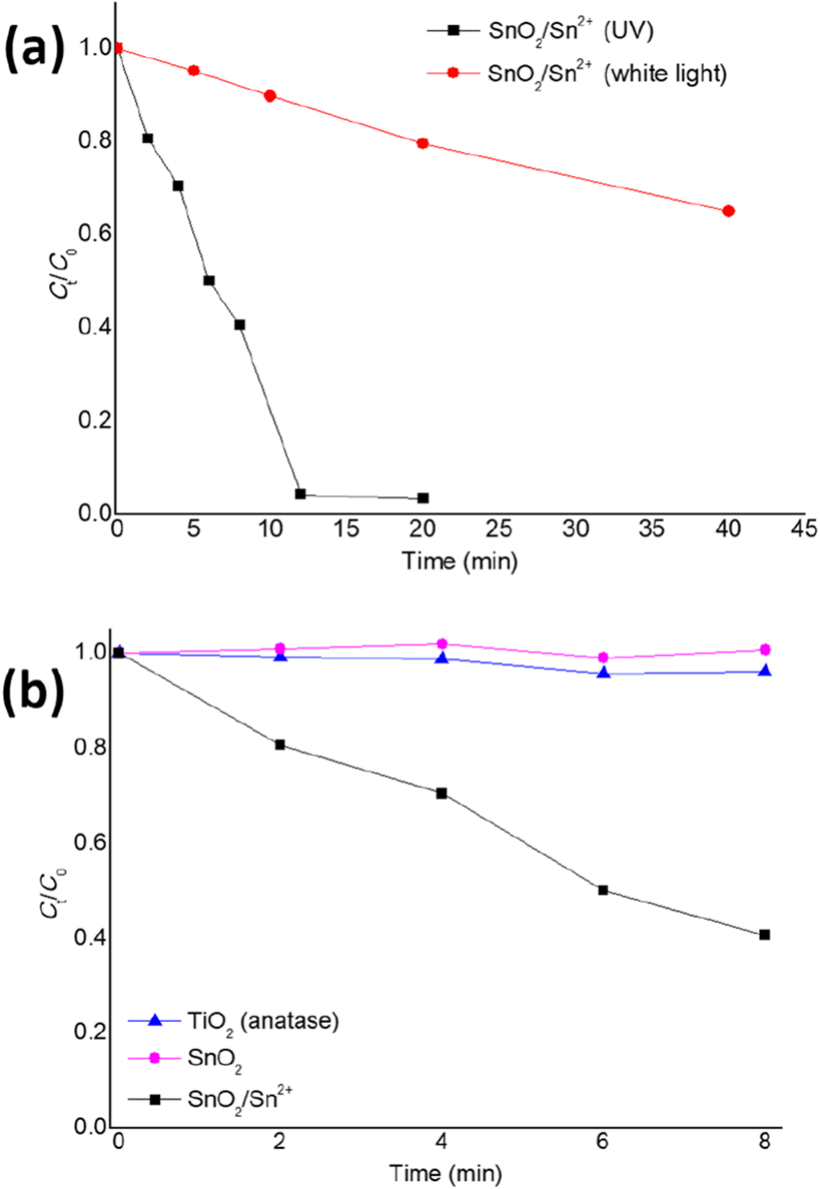
Photocatalytic discoloration of a 10 mg L^–1^ MO dye solution under UV and visible light irradiation at a photocatalyst dose of 0.40 g L^–1^ of the SnO_2_/Sn^2+^ material (**a**). In (**b**), it is possible to find the results from the tests with anatase TiO_2_ and SnO_2_.

Considering the potential application of the synthesized material in photocatalysis, its photocatalytic performance was tested in terms of the photodegradation of MO dye, a persistent pollutant model in wastewaters. Figure 7a compares the effect of light presenting different spectral emissions on the photocatalytic discoloration of a 10 mg L^–1^ MO dye solution. The adsorption equilibrium was attained after 90 min of contact in the dark, and ~ 10% of the MO content was adsorbed on the SnO_2_/Sn^2+^ material, as demonstrated in Supplementary Fig. S13a. No noticeable degradation via photolysis occurred, as shown in Supplementary Fig. S13b.

According to Fig. 7a, the SnO_2_/Sn^2+^ material manifested a high photocatalytic activity under UV light, reaching 97% of MO degradation in 12 min. Although with a lower photodegradation performance, the material proved to be photosensitive to visible light (Fig. 7a). The photodegradation of MO under UV irradiation was repeated for a photocatalyst dose of 5.33 g L^–1^, and the decomposition of the MO content nearly reached completion in 6 min (Supplementary Fig. S13c). The results from the LC–MS/MS analysis demonstrated that no photodegradation by-products were detected after the photodegradation of MO, indicating that its complete decomposition was attained (Supplementary Fig. S14a–c).

For comparison, the performance of the photocatalytic degradation of MO dye was further tested using anatase TiO_2_ (the most studied material in heterogeneous photocatalysis) and commercial SnO_2_. Figure 7b shows that both materials exhibited poor photocatalytic activity under UV irradiation, when compared to the SnO_2_/Sn^2+^ material’s efficiency (it is worth noting that black light lamps provide predominantly UVA, a less energetic component within the UV spectral range). These results indicated that the incorporation of Sn^2+^ ions in SnO_2_ associated with a relatively large surface area increased the photocatalytic activity of the synthesized material dramatically, being able to outperform TiO_2_ at less energetic irradiation conditions.

## Conclusions

A mixed-valence tin oxide material was synthesized via simple hydrothermal method. The material presented a polycrystalline nature down to nanoscale. Its particles exhibited a well-defined microrod morphology, indicating that they were, in fact, mesocrystalline and grew through nucleation followed by crystal growth (in the same manner as a monocrystal). It was verified that the microrods possessed a significant amount of Sn^2+^ cations residing within the grain boundaries. The investigation on the material’s crystalline structure indicated that the material could be tetragonal rutile SnO_2_, however, the abundant Sn^2+^ defects may have distorted the originally tetragonal crystalline structure of SnO_2_ into a monoclinic or triclinic crystalline structure. Furthermore, differently of SnO_2_, the synthesized material absorbed visible light very efficiently due to the insertion of Sn^2+^ states within its band gap structure, harnessing photons with wavelengths up to nearly 700 nm. Photodegradation experiments indicated that the material is able to decompose the azo-dye methyl orange, a pollutant model in water, under either UV or visible light, thus exhibiting promising photocatalytic properties to undertake the decontamination of waters and wastewaters under solar light. The photodegradation efficiency of the Sn^2+^-containing SnO_2_ material is substantially higher when compared with those of commercial TiO_2_ (anatase) and SnO_2_. Its superior photocatalytic performance is probably due to the efficient charge separation in its band structure as well as to its relatively high surface area (93.0 m^2^ g^–1^).

### Supplementary Information

Supplementary Figures.

## Data Availability

The datasets generated during and/or analysed during the current study are available in the Mendeley Data repository, https://data.mendeley.com/datasets/n6tr9f785f/1.
