# Supplementary material for: Assessment of the superior photocatalytic properties of Sn2+-containing SnO2 microrods on the photodegradation of methyl orange
Source: Sci Rep. 2023 Sep 7;13:14774. doi: 10.1038/s41598-023-40659-8 (PMC10485244; doi:10.1038/s41598-023-40659-8)
Supplement: Supplementary file 1 — Supplementary Figures. [file 41598_2023_40659_MOESM1_ESM.pdf]

Supplementary Material to the paper *Assessment of the superior photocatalytic properties of Sn^{2+}-containing SnO_2 microrods on the photodegradation of methyl orange*

Alexandre de Oliveira Jorgetto[1]*, Maria Valnice Boldrin Zanoni[2], Marcelo Ornaghi Orlandi[1]

[1] Department of Engineering, Physics and Mathematics, São Paulo State University (UNESP), Araraquara, SP 14800-060, Brazil.

[2] Unesp, National Institute for Alternative Technologies of Detection, Toxicological Evaluation and Removal of Micropollutants and Radioactives (INCT-DATREM), Institute of Chemistry, São Paulo State University (UNESP), P.O. Box 355, Araraquara, SP 14800-900, Brazil.

* corresponding author: alexandre.jorgetto@unesp.br

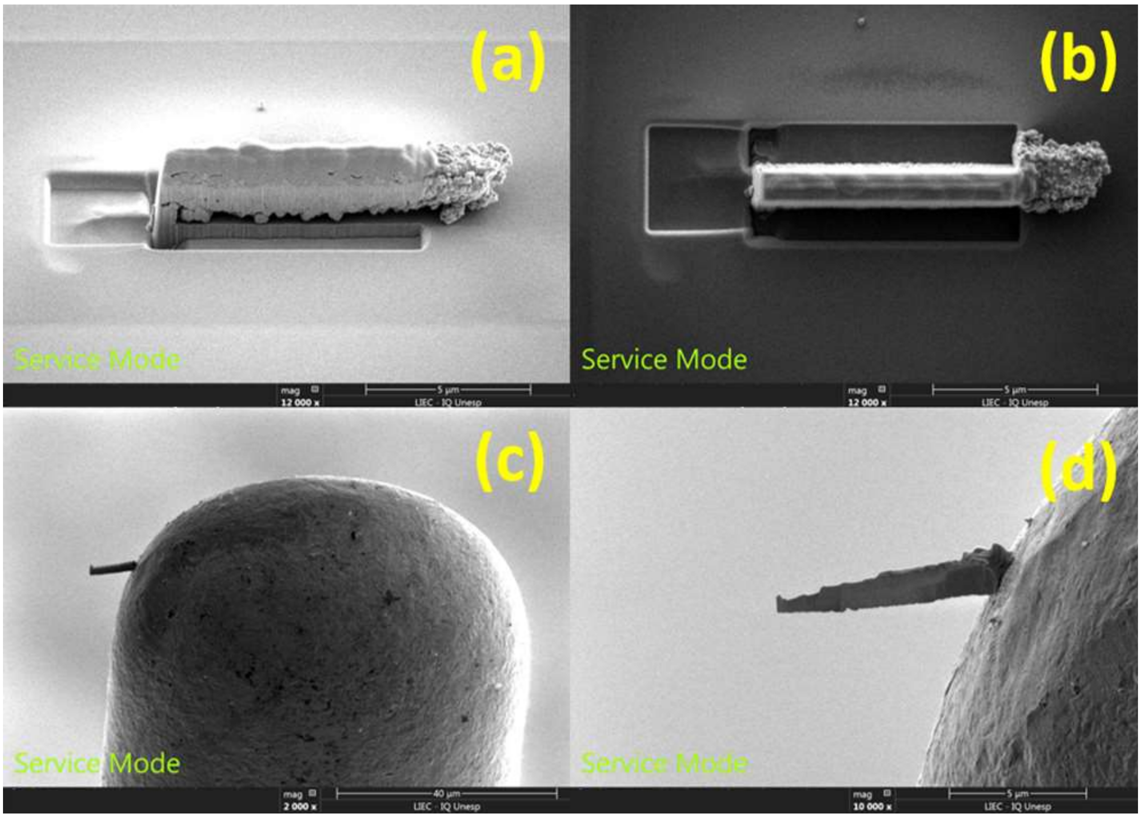

Figure S1. Lateral **(a)** and top **(b)** perspectives of the SnO_2/Sn^{2+} microrod thinned out in the FIB microscope. It is possible to notice the protective layer of carbon and platinum on the surface of the microrod. Image **(c)** shows the section of the microrod welded on a FIB lift-out sample holder, which was further thinned out with the ion beam **(d)** prior to the HR-TEM analysis.

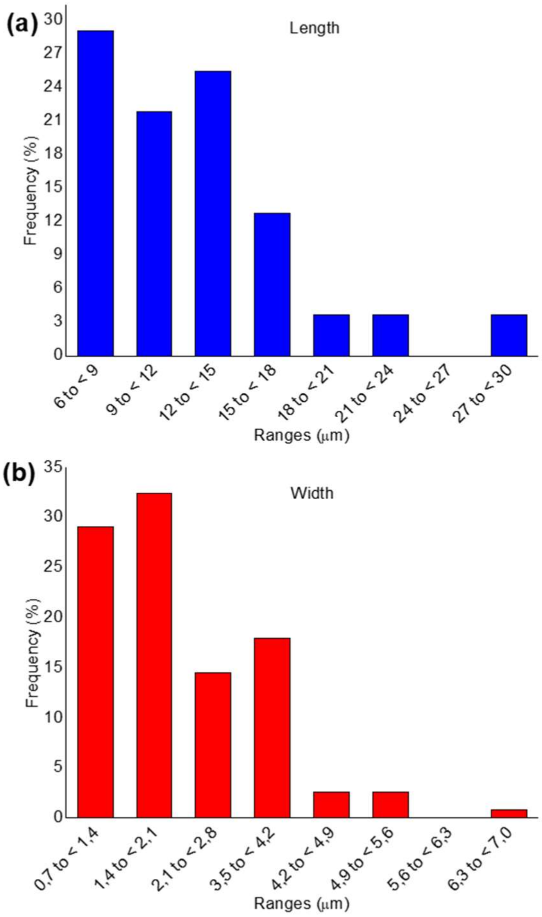

Figure S2. Histograms exhibiting the percent distribution of particles in terms of length **(a)** and width **(b)** of the microrods.

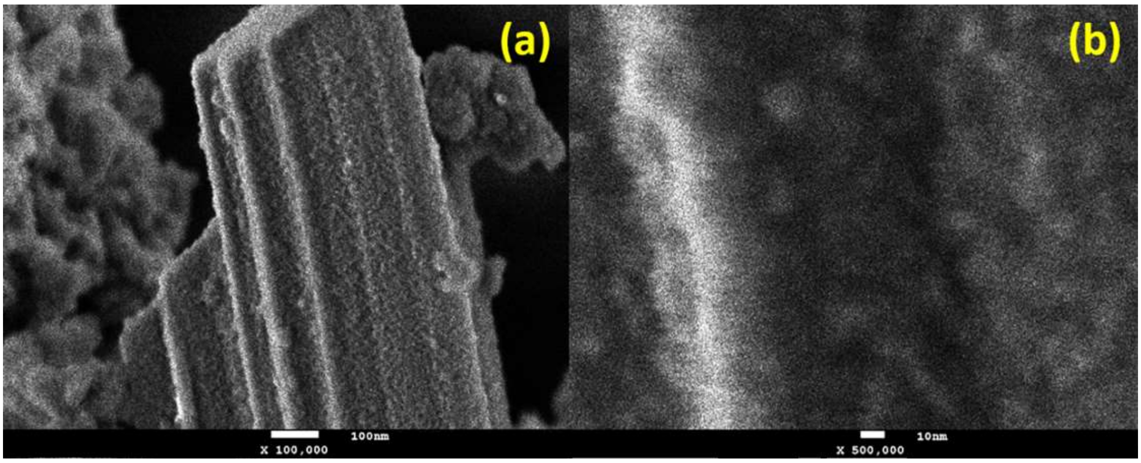

Figure S3. SEM images of a “smooth” surface SnO_2/Sn^{2+} microrod amplified 100 k× **(a)** and 500 k× **(b)**.

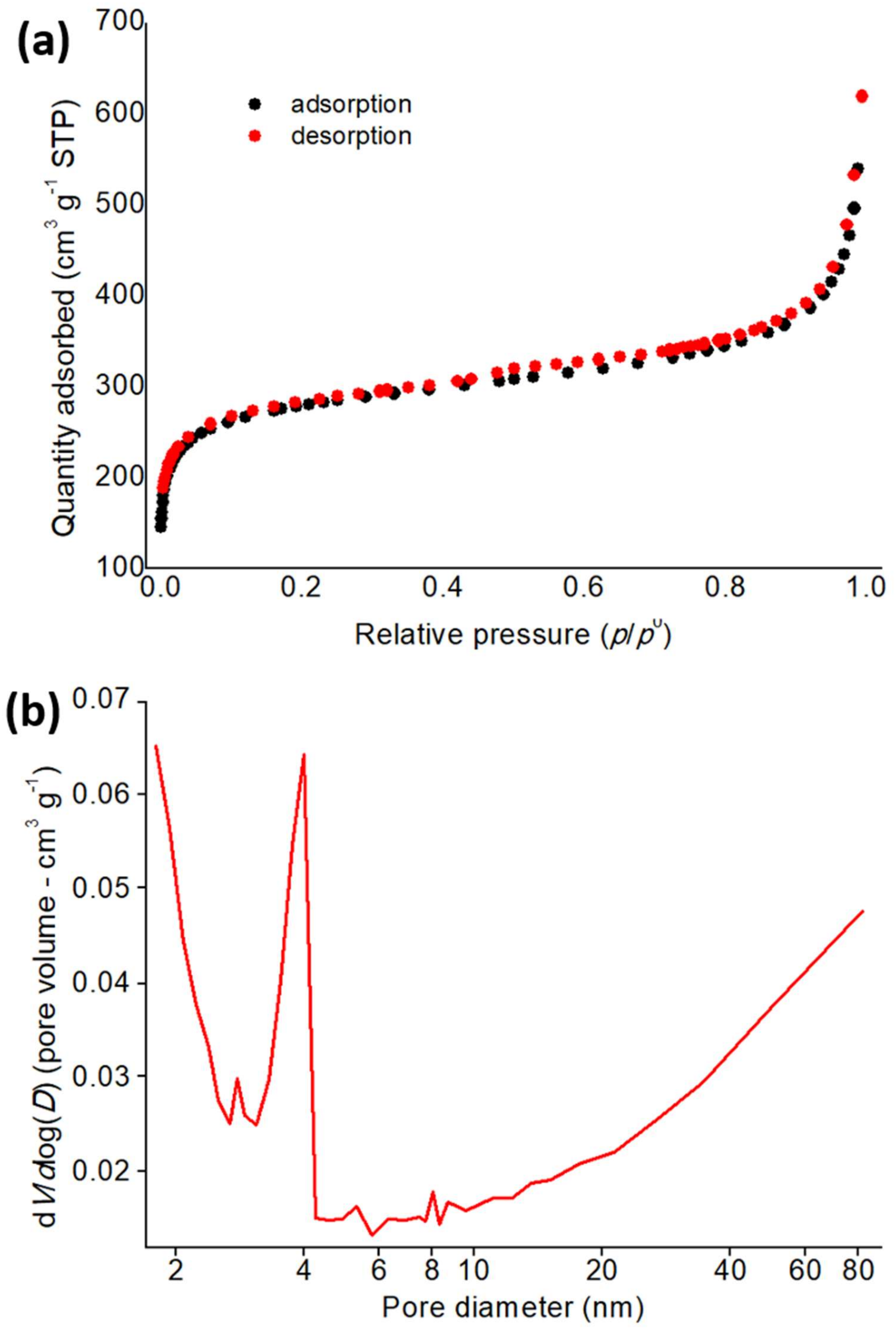

Figure S4. Nitrogen adsorption and desorption isotherms collected for the material SnO_2/Sn^{2+} **(a)** and the determination of the pore diameter distribution **(b)**.

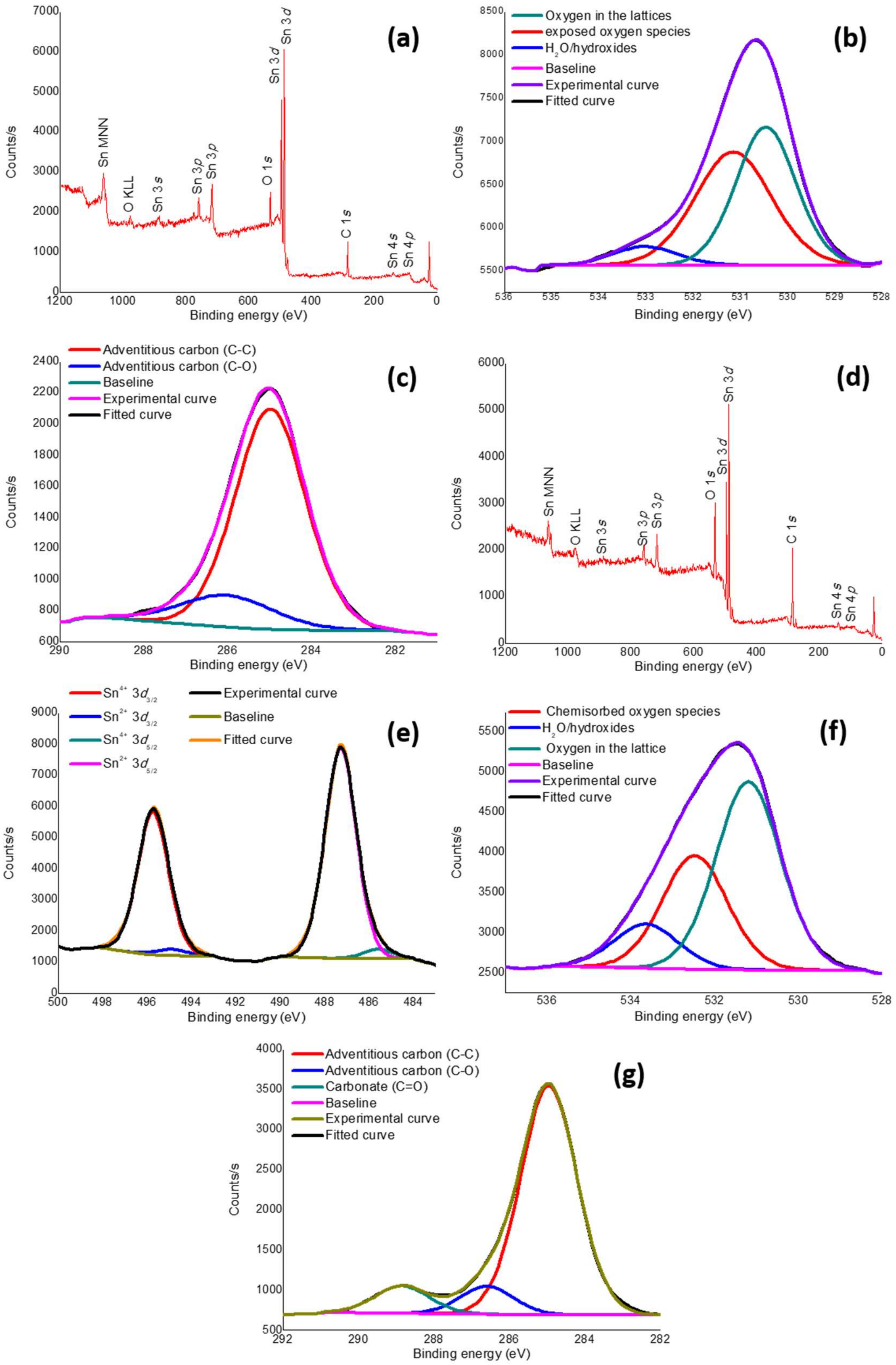

Figure S5. Results of the XPS analysis exhibiting the survey **(a)** and high-resolution spectra of the peaks of O 1*s* **(b)** and C 1*s* **(c)** of the sputtered SnO_2/Sn^{2+} material, and the survey **(d)** and high-resolution spectra of the peaks of Sn 3*d* **(e)**, O 1*s* **(f)** and C 1*s* **(g)** of the non-sputtered SnO_2/Sn^{2+} material.

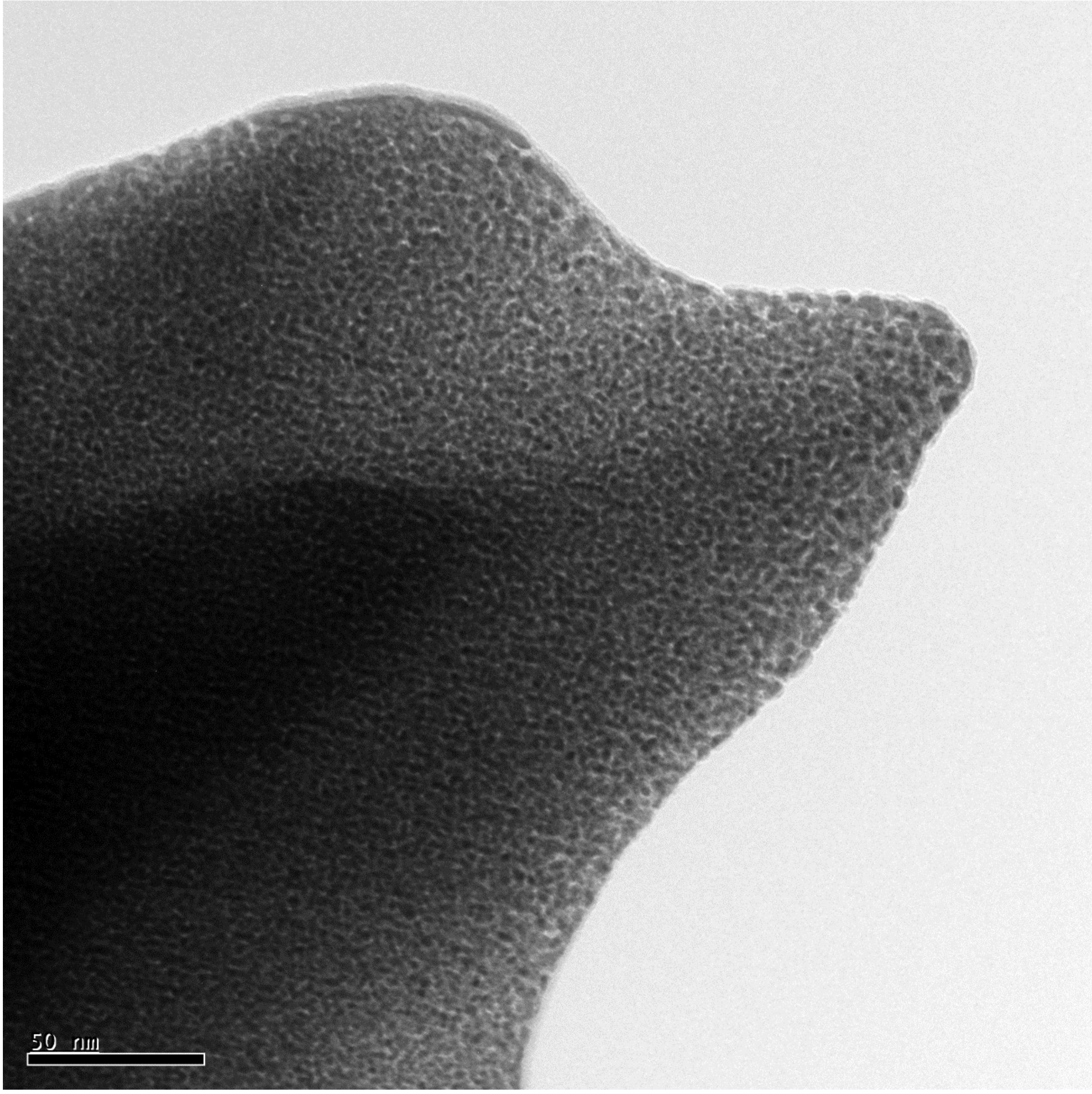

Figure S6. Bright-field TEM image showing the interior of a SnO_2/Sn^{2+} microrod.

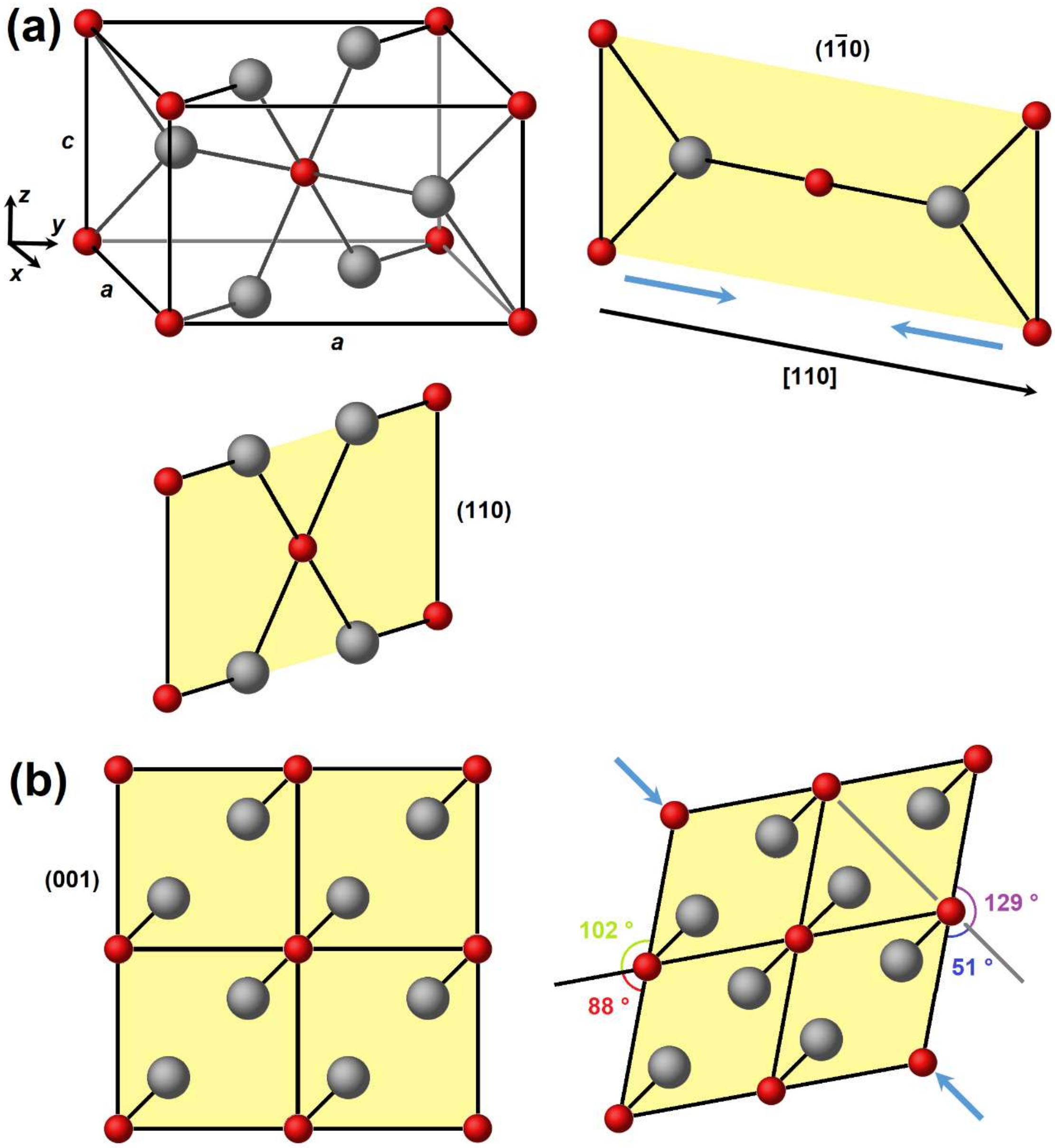

Figure S7. Unit cell of the tetragonal crystalline structure of rutile SnO_2 along with the isolated planes (110) and (1–10) **(a)**, and a scheme for a 10-% contraction of the SnO_2 unit cell along the direction [110], as seen from the top of the plane (001) and indicated by the blue arrows **(b)**. The contraction of the SnO_2 unit cell would induce the distortion of its originally 90 ° angles (structure on the left) to the angles exhibited in the crystalline structure on the right. In this figure, the dimensions are not to scale and the angles are exaggerated for clarity of perception.

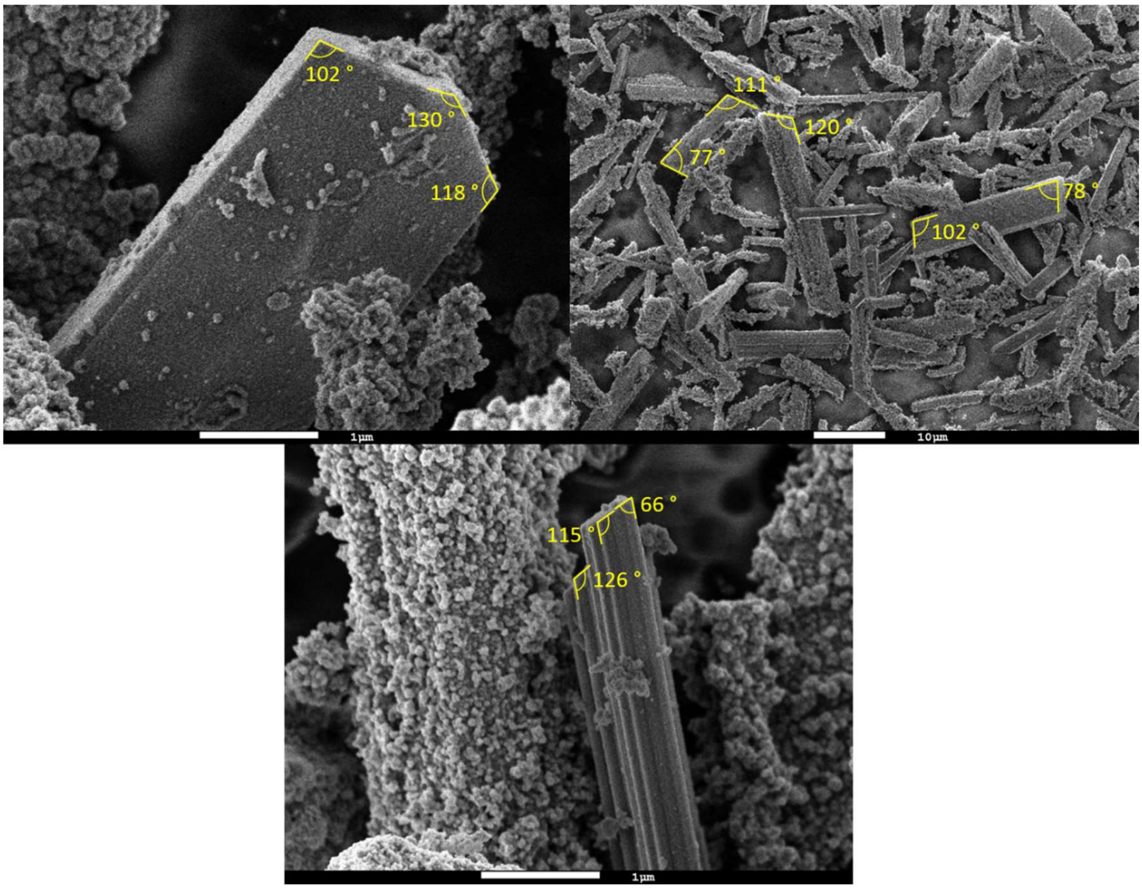

Figure S8. Determination of the angles in some SnO_2/Sn^{2+} microrods.

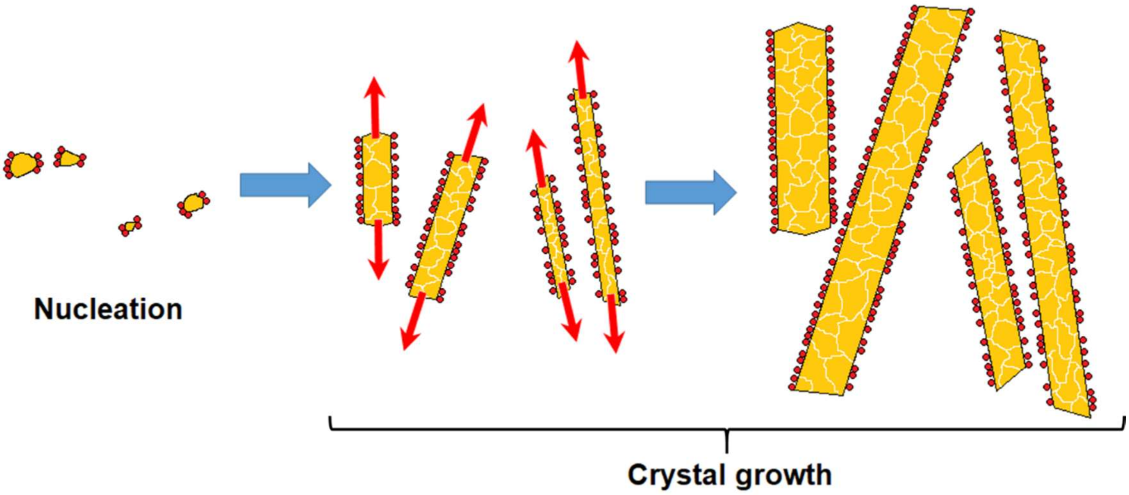

Figure S9. Schematic representation of the nucleation and growth of the microrods. The red circles represent the citrate molecules adsorbed preferentially on the lateral facets of the microrods, posing an inhibitory effect to their growth in this direction. The red arrows represent the growth direction of the microrods, less inhibited by the citrate molecules. White lines within the structures of the microrod demonstrate the grain boundaries forming during the crystal growth, yielding a mesocrystalline material.

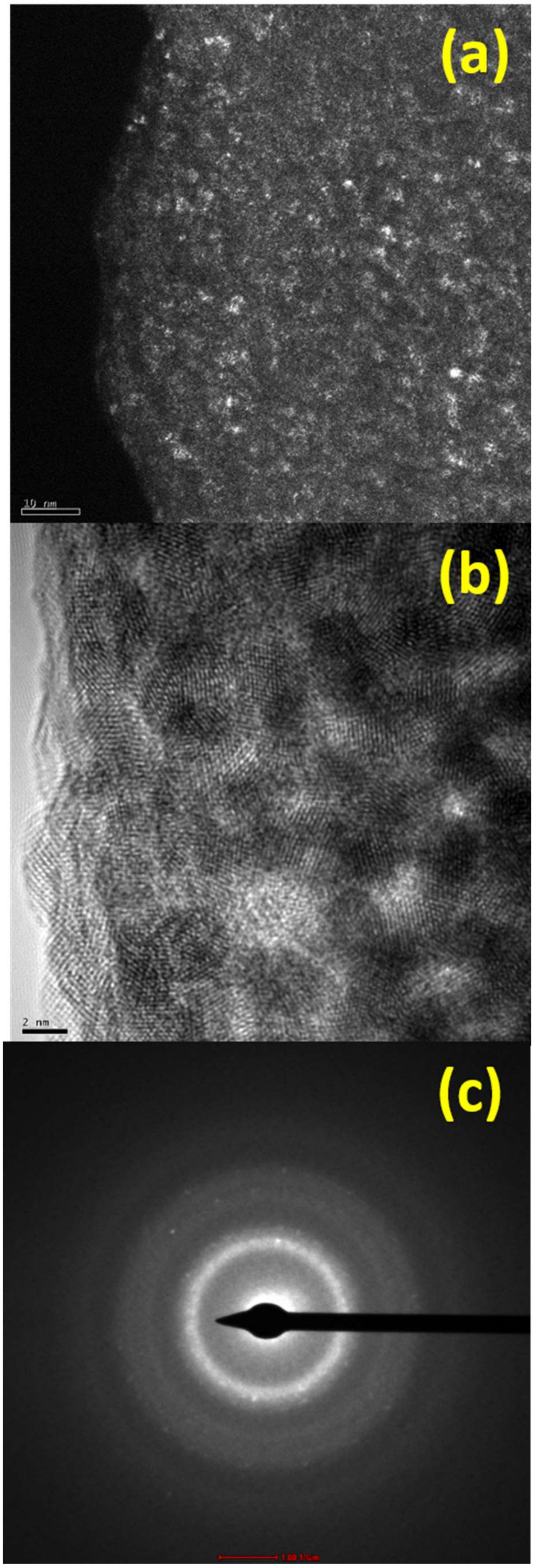

Figure S10. Dark field TEM **(a)** and HR-TEM **(b)** images along with the SAED analysis **(c)** of the internal structure of a SnO_2/Sn^{2+} microrod collected at its subsurface.

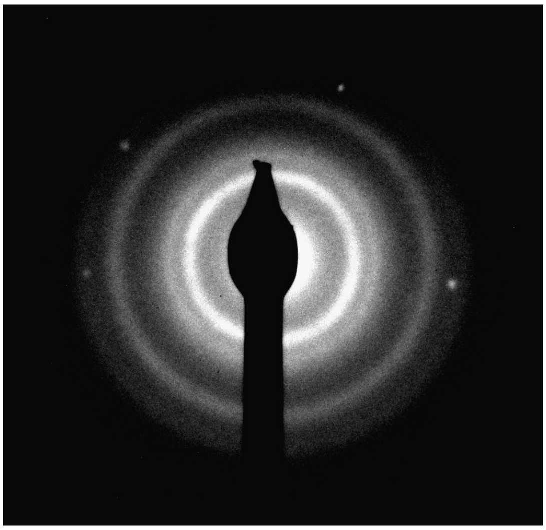

Figure S11. SAED analysis of a single nanosphere attached to the surface of a SnO_2/Sn^{2+} microrod.

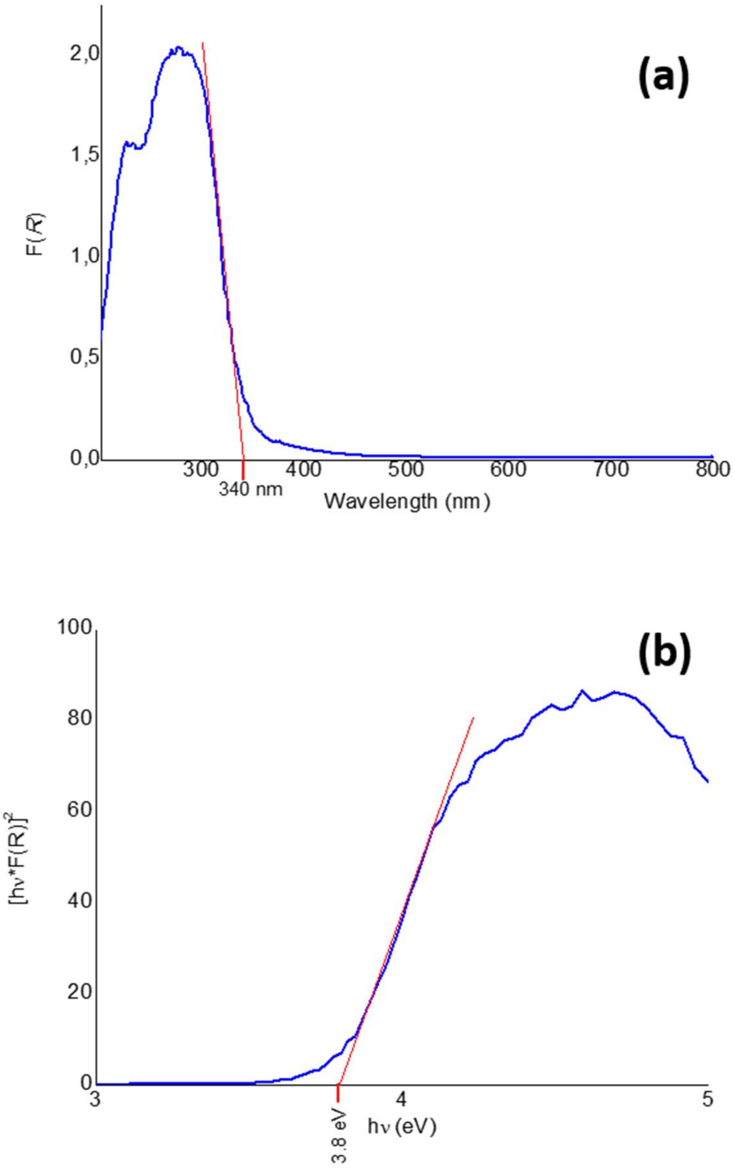

Figure S12. Results of the UV-Vis DRS analysis carried out for SnO_2 annealed at 1000 °C in air for 2 h, exhibiting its Kubelka-Munk **(a)** and Tauc **(b)** plots.

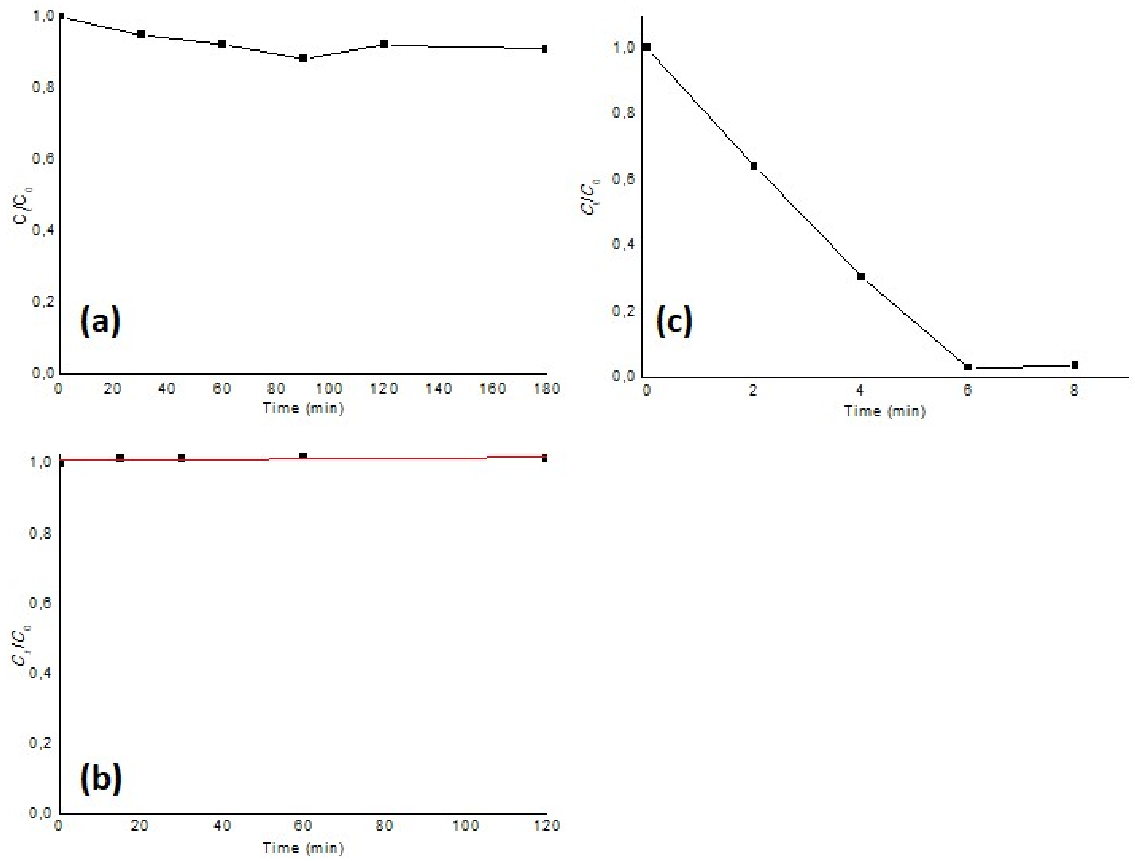

Figure S13. Adsorption test (dark regime) **(a)**, photolysis test without material **(b)** and photodegradation of MO at a photocatalyst dose of 5.33 g L^{-1} **(c)**.

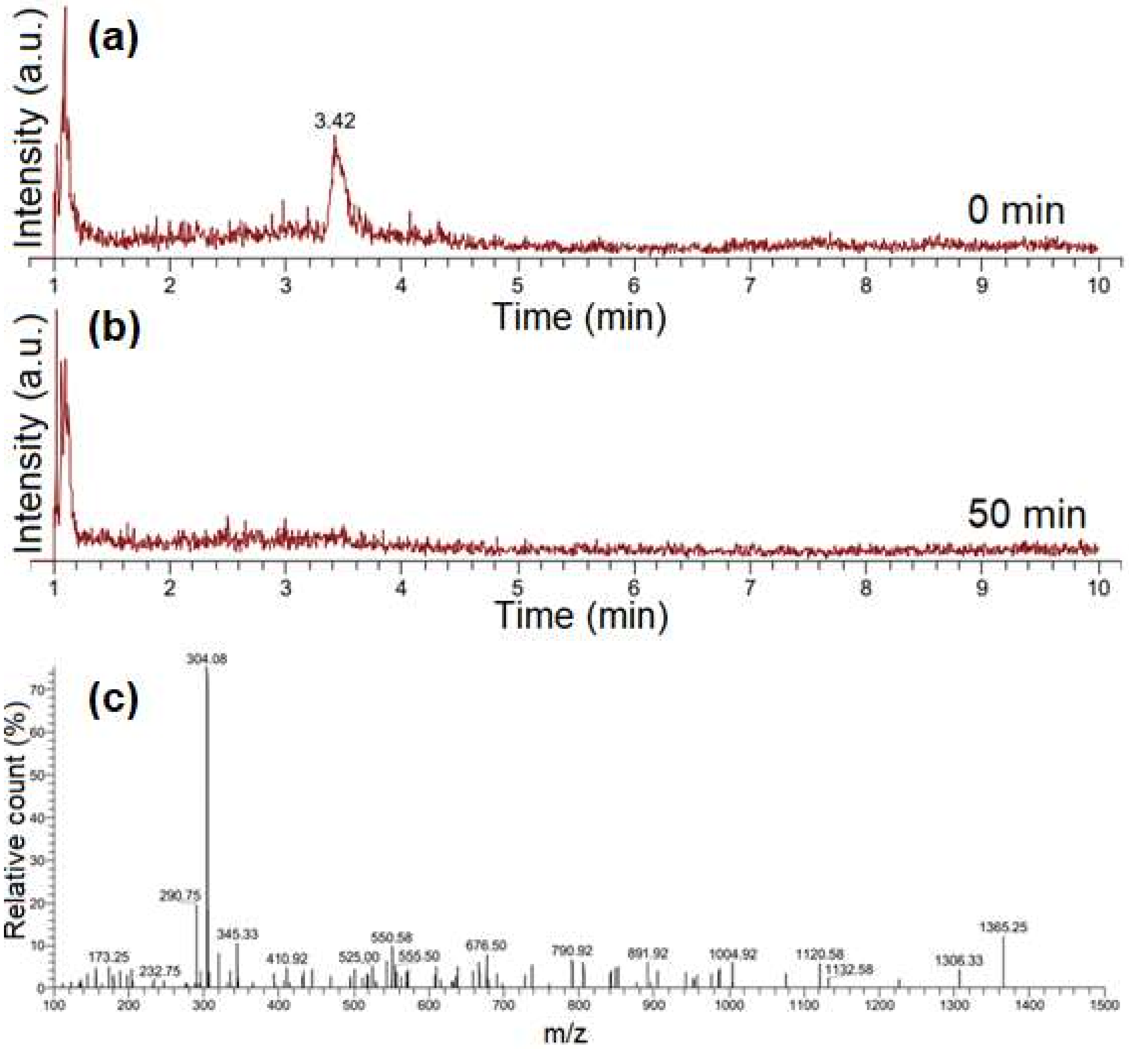

Figure S14. Chromatograms of samples collected at time 0 min **(a)** and after 50 min **(b)** of photodegradation with the material SnO_2/Sn^{2+}. The peak at 3.42 min in the first chromatogram correspond to the fragment *m*/*z* = 304.08 **(c)**, relative to the anionic structure of the MO.

In the chromatogram of Fig. S14 **(a)**, it is possible to notice a peak at the retention time of 3.42 min. The mass spectrum of this peak is expressed in Fig. S14 **(c)**, which demonstrated that the main contributor to this peak is the fragment of *m*/*z* = 304.08, associated to the molecular ion peak of the methyl orange anion (excluding the sodium mass). As can be noted from Fig. S14 **(b)**, the peak associated to MO disappeared completely for the sample illuminated for 50 min, and no other peak appeared, indicating that no by-products were generated after the photodegradation process using the material SnO_2/Sn^{2+} as photocatalyst.
